# Successful Aging for Community-Dwelling Older Adults: An Experimental Study with a Tablet App

**DOI:** 10.3390/ijerph192013148

**Published:** 2022-10-13

**Authors:** Beenish Moalla Chaudhry, Dipanwita Dasgupta, Nitesh V. Chawla

**Affiliations:** 1 School of Computing and Informatics, University of Louisiana at Lafayette, 104 E. University Circle, Lafayette, LA 70501, USA; 2 Department of Computer Science and Engineering, University of Notre Dame, Indiana, IN 46656, USA

**Keywords:** older adults, goal-oriented care, connected health, mobile application, human-centered design

## Abstract

Mobile health (mHealth) technologies offer an opportunity to enable the care and support of community-dwelling older adults, however, research examining the use of mHealth in delivering quality of life (QoL) improvements in the older population is limited. We developed a tablet application (eSeniorCare) based on the Successful Aging framework and investigated its feasibility among older adults with low socioeconomic status. Twenty five participants (females = 14, mean age = 65 years) used the app to set and track medication intake reminders and health goals, and to play selected casual mobile games for 24 weeks. The Older person QoL and Short Health (SF12v2) surveys were administered before and after the study. The Wilcoxon rank tests were used to determine differences from baseline, and thematic analysis was used to analyze post-study interview data. The improvements in health-related QoL (HRQoL) scores were statistically significant (V=41.5, p=0.005856) across all participants. The frequent eSeniorCare users experienced statistically significant improvements in their physical health (V=13, p=0.04546) and HRQoL (V=7.5, p=0.0050307) scores. Participants reported that the eSeniorCare app motivated timely medication intake and health goals achievement, whereas tablet games promoted mental stimulation. Participants were willing to use mobile apps to self-manage their medications (70%) and adopt healthy activities (72%), while 92% wanted to recommend eSeniorCare to a friend. This study shows the feasibility and possible impact of an mHealth tool on the health-related QoL in older adults with a low socioeconomic status. mHealth support tools and future research to determine their effects are warranted for this population.

## 1. Introduction

Most conceptualizations of Successful Aging emphasize the avoidance of disability and high levels of physical functioning as requirements for well-being. For example, Rowe and Kahn’s [1] Successful Aging model is based on, (a) the absence disease and related disability, (b) high cognitive and physical functioning, and (c) active engagement with life. There are two major limitations of these models. Firstly, they fail to include older adults who self-report aging successfully despite their chronic conditions or disabilities [2]. Secondly, they fail to acknowledge that chronic conditions and disabilities are almost unavoidable with age. Research indicates that in their late 60s, as many as 87.6% of the individuals have at least one chronic condition [3], and at least 45% experience two or more [4]. This means that less than 12.4% of older adults in the United States can be classified as aging successfully. To address this issue more broadly, in this research, we use newer models of Successful Aging [5,6] that recognize disabilities due to age, and incorporate management of chronic conditions and disabilities along with the maintenance of physical and mental health, and active social engagement as its main components [7]. This allows us to understand Successful Aging through the lens of a person’s Quality of Life (QoL), which can be defined simply as “the degree to which an individual is healthy, comfortable, and able to participate in or enjoy life events” [8].

For older adults (≥65 years old) with lower socio-economic status (SES) [9] (i.e., low-income and low literacy), systematic barriers make even this nuanced conceptualization of Successful Aging a challenging endeavor [10]. An examination of socio-economic factors explains how people with low-educational attainment and low-income can fail to meet the objective measures of Successful Aging. Low literacy leads to low health literacy, which often implies unhealthy lifestyles, more chronic conditions (hypertension, diabetes etc.), and higher mortality rates [11,12]. Inadequate income makes appropriate medical care unattainable, which can increase the chances of disability and lower the quality of life. In addition, there is a strong link between low-income and low literacy because low-wage jobs often have low educational expectations. A strong positive correlation is also known to exist between lower SES and poor mental health [13]. Effective strategies, therefore, must be devised to help older adults, particularly those with lower SES, to take charge of their own health and age successfully [14].

With the ubiquity of mobile technologies such as smartphones and tablet PCs, mobile health (mHealth) applications that address various aspects of Successful Aging have started to emerge. For example, mHealth apps have been developed to help individuals with medication management [15,16], symptom monitoring [17] and activity tracking (e.g., maintenance of physical health) [18,19]. Despite the abundance of such technologies, recent scoping reviews reveal several shortcomings of the existing work [20,21]. The major limitation is that the existing studies use mHealth apps aimed at only one aspect of Successful Aging such as social engagement [22] or physical health [23], while focusing on a specific group of older adults, that is, those who were independent and wealthy [24]. Moreover, most published manuscripts are solution proposals [25,26], and validated in controlled experiments or usability studies using a specific sample of older adults [27]. It remains to be observed whether mHealth apps are effective in simultaneously promoting more than one aspect of Successful Aging, especially in older adults with lower SES (i.e., low income and low literacy skills) [28].

To address these gaps, we collaborated with a community health program at a local hospital in the United States to develop a tablet application that targets two main components of successful aging, i.e., physical and mental health. The eSeniorCare app was built using an iterative, human-centered design approach [29,30,31,32,33,34] involving the target population and their care providers over a course of five years (2014–2019). In this paper, we describe a 24-week pilot trial to test the feasibility of the eSeniorCare in community-dwelling older adults with low SES. Twenty five participants (female =14), with a mean age of 65 years and having at least one chronic condition, from two low-income independent living facilities voluntarily participated in the study. The participants were expected to use the application in their daily life. Our contributions include: (a) description of an mHealth intervention for promoting Successful Aging in older adults, (b) evaluation of the feasibility of an mHealth app in community-dwelling older adults with low SES, and (c) description of the challenges of implementing a technology-based solution for promoting Successful Aging in the target population.

In the next section, we describe the study methods, tablet intervention, study participants, evaluation metrics, and data analysis techniques. Section 3 describes the quantitative and qualitative findings based on the collected data. We, then, discuss the implications of the study findings through a comparison with the published literature. We close the paper by highlighting the study’s limitations and presenting the conclusions.

## 2. Materials and Methods

The primary aim of this study was to test the feasibility of a tablet-based mHealth app in community-dwelling older adults with low SES to improve self-perceptions of Successful Aging. A substantial number of secondary outcomes were included to facilitate the understanding of this primary outcome, including changes in self-ratings of physical health, mental health and health-related quality of life, perceived benefits and barriers of tablet-based applications, and acceptance of technology for the self-management of health and wellness.

### 2.1. Study Design

We conducted this study in collaboration with the Aging-in-Place (AiP) program offered by the Community Health Enhancement Initiative of The Memorial Hospital, South Bend, Indiana. One staff member of the AiP program, called the Resident Life and Health Administrator (RLHA), helped us recruit participants and coordinate all aspects of the study. The ethical review boards of The University of Notre Dame and The Memorial Hospital, South Bend, Indiana (Protocol # 13-04-1048) gave us the approval to conduct the study at two local independent living facilities, where AiP was providing its services free of charge at the time of the study. According to the rules of the independent living facilities, the residents were required to be 65 years and above and living below the poverty line (annual income ≤10 K for one person). Individuals aged between 55–64 years were also allowed to reside in these facilities if they were on disability aid because of existing chronic conditions. Therefore, in this study we recruited individuals aged ≥65 years and also 55–64 years old individuals with existing chronic conditions. By virtue of being in an independent living situation, residents lived separately, but also enjoyed the company of their peers. The goal of the AiP program was to accommodate the housing, transportation, health and social needs of the residents, and to support healthy and successful aging in place by offering progress such as exercise classes, seminars, and services, etc.

To recruit participants for the study, we conducted two meetings at the approved study locations. During the meetings, we invited the residents to participate in the study by explaining the study purpose and inclusion criteria (described below). At the conclusion of the meeting, interested residents voluntarily signed up to participate in the study. The interested residents were then invited to a follow-up meeting, where they were first informed of their rights as study participants and privacy information regarding the collected data. The residents who agreed with these procedures signed a consent form. Following this, participants were screened and enrolled if they met the following inclusion criteria: mild to no cognitive disability (determined by the Saint Louis University Mental Status Exam (SLUMSE) [35]), fluency in English, and good vision. SLUMSE was used as the screening tool because it was been used by the AiP to screen participants for dementia. Participants were excluded if they were planning to move out of the independent living facilities, uninterested in study activities, or if they had cognitive impairments. Participants who passed the screening and met the eligibility criteria completed a demographic information form. They were, then, asked to complete the pre-study assessments described in Section 2.5.

The study was based on single-arm experimental pre–post design, which allows researchers to measure variables of interest both before and after an intervention is implemented in the same participants [36]. It is typically used when the goal is to obtain preliminary evidence but not the confirmatory evidence of the efficacy of an intervention [37]. The intervention consisting of the eSeniorCare app and tablet games was provided to the participants on a password-protected, encrypted Android tablet. Participants were informed that they would own the tablet upon completion of the study. The participants were expected to use the eSeniorCare app to self-manage their medications and health goals, in collaboration with the RLHA who was responsible for providing relevant services to residents under the AiP program. In addition, we expected them to play the assigned tablet games.

The study was originally planned for 12 weeks; however, due to the technical issues and participants’ training needs, we ended up running the study for 24 weeks plus 12 weeks of technology workshops. At the conclusion of the study, participants completed the same assessments (Section 2.5) that they had completed at the beginning of the study. In addition, they participated in a 45-min semi-structured interview with the researchers. The purpose of the semi-structured interview was to understand how participants used the application, and the benefits and challenges they experienced as a result of using it. Participants completed the assessments and interviews voluntarily. They were free to skip any assessment items or interview questions that they did not want to answer.

### 2.2. Technology Training and Support

Before the study began, we held technology workshops to train the participants to operate the tablet and use the assigned apps. A 60-min workshop was held every week at each building from where the participants were recruited for three months. The specific workshop topics and number of times each topic was covered are listed in Table 1. Paper manuals on these topics were developed and distributed among the participants. Each workshop was run by 2–3 instructors and three peer mentors. Further details about the workshops have been given in the related manuscript [38].

During each workshop, participants were seated in groups of five for efficient management and targeted training. The tutors presented the workshop topic using a PowerPoint presentation or hands-on demonstrations on a tablet. The participants were then expected to practice the demoed topic on their own. They also could seek help from the instructors and peer mentors, and consult the paper manuals. At the end of the workshop, if participants felt prepared, they could demonstrate competency by completing the tasks in front of the workshop facilitator. Otherwise, they could do so in the next workshop or whenever they were ready. In general, a participant used 3–4 tries to demonstrate competency in the workshop tasks. Twelve workshops were held at each site. No participant attended all the workshops, but 11–15 participants were present at each. On average, everyone attended at least eight workshops.

### 2.3. Study Flow

**Figure 1:**
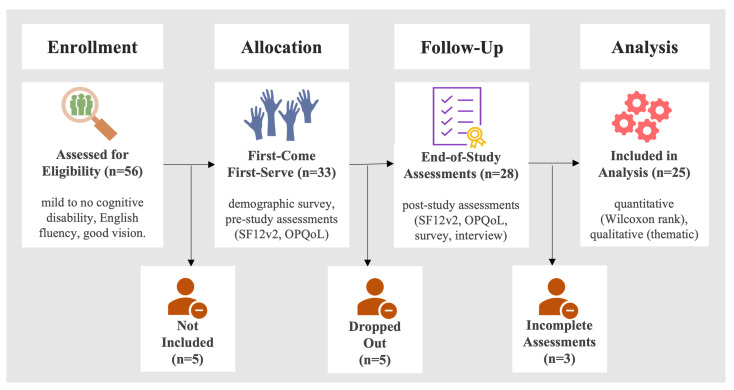
Participant flow diagram.

A total of 56 residents demonstrated interest in participating by signing up at the end of the study announcement. These residents were then invited to the screening meeting, where informed consent and SLUMSE were administered. Fifty one participants met the inclusion criteria and were sent an invitation letter to attend the pre-study assessment sessions. Thirty three participants completed the pre-study assessments and were assigned a tablet with eSeniorCare intervention installed. During the actual study, two participants moved out of the independent living facilities and, hence, dropped out of the study. Two more participants discontinued due to unspecified reasons. Another participant did not continue the study because of a death of a family member. The remaining 28 participants continued the study, without officially quitting. However, three participants did not complete the post-study questionnaires. Therefore, they were excluded from the analysis. The attrition rate was less than 25 percent, which is lower than the rate reported in earlier studies that have been conducted for similar purposes. Hence, 25 participants completed the study and filled out all the questionnaires at the end of the study. The participant flow has been summarized in Figure 1.

### 2.4. Participants

In this paper, we present our findings for 25 participants (11 males and 14 females) who completed the study and completed all the questionnaires (Table 2). The age of the participants ranged from 56 years to 83 years (mean = 65, SD=7). Twenty two participants identified themselves as African Americans. Nineteen participants reported an income of less than $10 K. Fifteen participants had between 13–15 years of formal education while ten had ≤12 years. Twenty participants were taking prescribed medications at the time of recruitment. Twelve participants had one chronic condition and the remaining participants reported having multiple chronic conditions. All but five participants lived alone. Seventeen participants rated themselves as novice users of technology. The remaining eight indicated that they felt comfortable using tablets because of their prior experience with tablets in a related study. No one owned a personal computer or a smartphone.

### 2.5. Tablet Intervention

The eSeniorCare system was developed using an incremental and iterative human-centered design approach [29,30,34]. It is meant to support and promote different aspects of Successful Aging, particularly physical and mental health by allowing older adults to self-manage their medication intake and health activities (through goal setting and tracking).

#### 2.5.1. Platform

The system consists of a mobile app, and a web portal. The mobile app was developed for Android platforms using Java, whereas the web portal was developed with PHP. Both applications were connected to a PHP-based REST API built to store and retrieve data from the central MySQL database. Security was enabled through the Lightweight Directory Access Protocol (LDAP) installations.

#### 2.5.2. Non-Functional Requirements

Non-functional requirements define a system’s operational capabilities and constraints. They are typically specified in terms of performance, safety, security, and quality attributes. The following non-functional requirements were devised for the eSeniorCare system.

(a) Performance Requirements
  - The applications should be ready for user interaction within 3 s of launch.
  - The interfaces should be updated within 2 s of user interaction.
  - The database should use normalization to eliminate data redundancy.
  - The application should upload new data from the user’s tablets whenever a Wi-Fi connection is available.
  - The users’ data from their mobile applications should be uploaded to the central database on a separate thread to prevent the disruption of normal user interactions during the upload.
(b) Safety Requirements
  - The data in the central database must be backed up every week.
(c) Security Requirements
  - All keys used for the REST API must be stored securely.
  - REST API should be the only method of connect to the central database.
  - The central database should be behind a firewall.
  - The applications must prompt users for password whenever they login.
(d) Software Quality Attributes
  - Availability: The AiP staff must be able to access their portal throughout the week at any time during the day. The system unavailability should not be more than one working day, even in the case of unplanned down time.
  - Usability: The interface should be easy to learn without a tutorial and should allow users to accomplish their goals without errors.
  - Customizable: The system should be flexible to accommodate user specific customizations.
  - Accessibility: The system must meet Web Content Accessibility Guidelines 2.1.

#### 2.5.3. Mobile App

The target users of the eSeniorCare mobile application were older adults. The software specifications for the app have been detailed below.

(a) Health Goals

**Figure 2:**
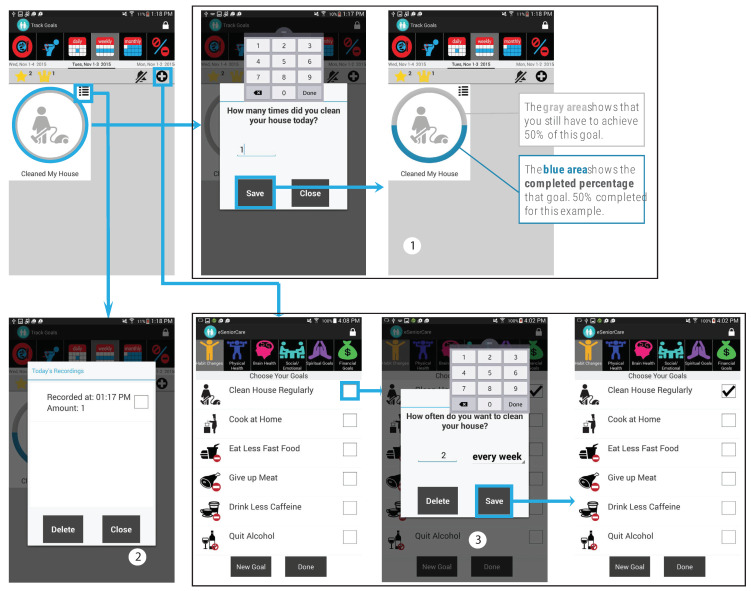
Features of eSeniorCare’s goal component. (**1**) Goal tracking process, (**2**) Viewing tracking history, and (**3**) Goal setting process.

Health behavior change strategies derived from various behavioral health theories are typically used in digital health interventions to encourage people to adopt healthy lifestyles [39,40]. Goal-setting [41] is one such strategy that encourages individuals to direct their efforts towards the aim that they want to achieve [42,43]. Therefore, we incorporated a goal-setting component within the eSeniorCare app to encourage participants to think about their health needs and create goals to meet those needs (Figure 2). Participants could choose the goals suggested by the app, or create new ones (Figure 2: Label 3).

To ensure that the goals were evidence-based and meaningful, participants were encouraged to either cross-check or set their goals in collaboration with the RLHA and/or their primary care providers. Participants can set both specific, measurable, attainable, realistic and time-bound (SMART), and non-SMART goals. The SMART goals help the RLHA monitor participants’ progress and decide what support they should provide to participants. ‘Play tablet games for 30 min every day’ is an example of a SMART goal, and ‘Meet my primary care provider this week’ is an example of a non-SMART goal. After setting goals, participants could track them according to the tracking frequency (once, daily, weekly, and monthly) and unit (amount of time and number of times) they had chosen when they had set the goal (Figure 2: Label 1).

We incorporated various features within the app to motivate the participants to achieve their target goals. Each goal is denoted by an icon, which is surrounded by a gray circle that turns blue (Figure 2: Label 1) to indicate the percentage of the goal target achieved by the participant. Participants received stars to meet their goals and crowns to overachieve them. They can review and delete any tracked data related to their goals (Figure 2: Label 2). The participants can also set reminders to remind themselves to achieve their goals, allowing the RLHA to monitor progress and, consequently, provide timely support.

The functional requirements of the goal-setting module are given as follows. The user should be able to:

- obtain goal suggestions,
- verify goal choices with the care providers,
- set SMART and non-SMART goals,
- track goal progress,
- set reminders about goal tracking,
- obtain help from the AiP staff,
- modify goal choices and settings.

(b) Medication Intake

**Figure 3:**
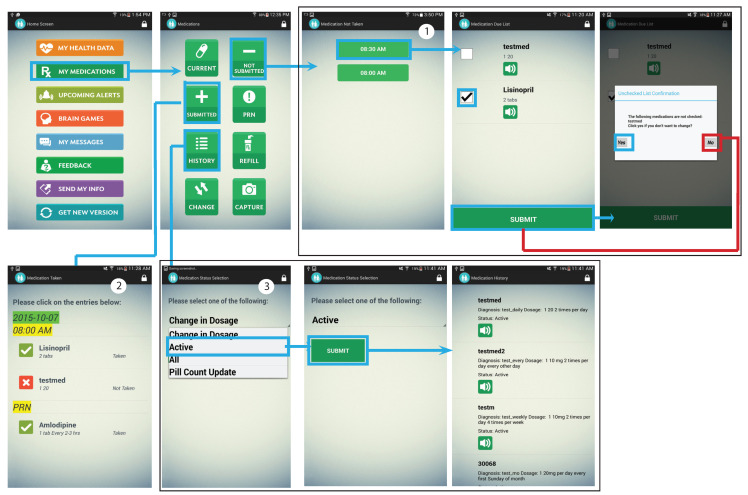
Features of eSeniorCare’s medication component. (**1**) Medication submission process, (**2**) Submitted medication, and (**3**) Subscription history.

The purpose of this component was to enable participants to manage and increase their adherence to prescribed medications. Each participant’s medication information (medication name, dosage, and intake time) was entered into the app by the RLHA via their web portal. The app generated medication intake reminders five minutes before a medication was due (Figure 3 Label 1). The reminder consisted of an alarm (i.e., sound) and a dialog box containing a list of medications due at the time, along with dosage information, images of the medications, audio buttons to hear medication name. A checkbox associated with each medication had to be checked to acknowledge the reminder and record the medication intake. However, if a medication was not checked, it was recorded as not being taken, and the non-compliant participant was added to the alert list on the RLHA’s portal. The RLHA then took appropriate steps to help the participants get back on track with medication intake.

The participants could also view their prescribed medications along with other details including the condition for which the medication was prescribed within the app, so that they could communicate effectively with healthcare providers during medical visits and prevent any reactions from further prescriptions. Any changes to the existing medication list were communicated to the RLHA, so she could make corresponding updates to the participant’s medication list on her web portal. Furthermore, the participants could view their medication history (filtered by status (Active; Discontinued) and dosage) in the application (Figure 3: Label 3, Figure 3: Label 2). Participants can also maintain a list of pro-re-nata (PRN) medications or medications taken as needed, along with their pictures in a separate feature of the app.

The functional requirements of the medication intake module are summarized below. The user should be able to:

- enter new medication information (including over-the-counter medication) information, such as medication name, photo, dosage, prescription date, etc.,
- receive medications intake reminders,
- review daily medication schedule,
- track medication count and record intakes,
- review medication intake history,
- request medication refills from the pharmacy,
- update prescription information and medication list.

#### 2.5.4. Web Portal

The app was connected to a web portal, where the RLHA could monitor the goal progress and medication intake patterns of each participant. In case of concerning behaviors, such as inconsistent medication intake or goal achievement, the RLHA could use the portal to send personalized messages to participants on their tablets. The RLHA could also receive messages from the participants and view them on their portals. This would help them resolve participants’ issues by providing timely help and guidance, thereby maintaining the continuity of care. The functional requirements of the RLHA portal were as follows:

The RLHA should be able to:

- review medication and goal tracking history of each participant,
- review new goal and medication entry requests from each participant,
- send messages (reminder or announcements) to each participants,
- read messages sent by each participant,
- create new participant accounts.
- receive medication and goal updates from each participant.

#### 2.5.5. Tablet Games

The purpose of this component was to provide opportunities for participants to engage in mentally stimulating activities such as games. Research suggests that puzzle games such as Jigsaw [44] and Word Search [45] produce positive changes in cognitive function including enhanced executive function, memory, and focused attention. Other evidence suggests that more traditional, analog games [46] such as card games [47], crosswords, and Sudoku [48] slow down the cognitive decline associated with aging. Based on this evidence and feedback collected from participants, we installed 10 puzzle games including Word Search, Solitaire, Jigsaw Puzzles, Tangram and Sudoku on participants’ tablets. Training on these apps was provided during the weekly technology workshops, and the participants were encouraged to play these games throughout the study. Additionally, the participants could download and play other games of interest from the Google Play Store.

### 2.6. Evaluation Questionnaires

We used the following questionnaires to evaluate the impact of the tablet application and games. Both provide a subjective assessment of Successful Aging:

#### 2.6.1. Short Form 12-Item (Version 2) Health Survey

The Short Form version 2 (SF12v2) health survey [49] is a 12-item validated questionnaire targeting eight functional domains: bodily pain, physical function, role physical, role emotional, mental health, vitality, social function, and general health. These domains are further classified as a physical component summary (PCS) and mental component summary (MCS), which correspond to self-assessments of physical and mental health, respectively. In essence, PCS is a combination of SF12v2 categories that primarily evaluate an individual’s general health, bodily pain, physical function, role physical, etc. On the other hand, MCS is a combination of SF12v2 domains that focus on individuals’ perceptions of depression, anxiety, and social activity. We administered this instrument both at the beginning and at the end of the study to assess participants’ perceptions of their health. We chose SF12v2 because, compared to SF36v2, it is both a concise (hence, reducing patient burden) and comprehensive patient-reported outcome measurement [50]. It has also demonstrated efficacy across age, disease and treatment groups [51,52]. In contrast, other surveys are either disease-specific (e.g., QLQ-C30 [53]), or domain-specific (e.g., Duke-UNC Functional Social Support [54]), which restricts their usage in the general population.

#### 2.6.2. Older Person’s Quality of Life

The Older Person’s Quality of Life (OPQoL) [55] is a 35-item validated questionnaire containing questions about: life overall, health (HRQoL), social relationships/leisure and social activities, independence, control over life, freedom, home and neighborhood, psychological and emotional well-being, financial circumstances, and religion/culture. Each question has a 5-point Likert response scale ranging from “Strongly Disagree” to “Strongly Agree”. The score in each dimension was calculated by summing the responses to related items [56]. We used this survey because it allows the measurement of several dimensions of quality of life (that AiP was interested in collecting for their own purposes) on a single scale, eliminating the need to use multiple scales that can increase the response burden on the participants. We administered this instrument both at the beginning and the end of the study. However, due to the nature of this study, we only report changes in HRQoL. It should be noted that there are overlaps in questions present in the OPQoL’s health dimension (HRQoL) and SF12v2’s PCS.

#### 2.6.3. Semi-Structured Interview

Towards the end of the study, we conducted a 45-min semi-structured interview, including a short survey with each participant to understand their overall experience with the eSeniorCare app. In particular, we asked participants to provide feedback on: (a) the perceived benefits of using the application; (b) their dislikes about the application; (c) their preference for: a tablet-based application versus a paper-based system for managing medications and activities, and why?, (d) whether they would recommend eSeniorCare to a friend?, and, finally, (e) suggestions for improving the eSeniorCare app. The interviews were voice-recorded and transcribed for analysis.

### 2.7. Data Analysis

Qualitative and quantitative data were collected during the study. We subjected the data to the following analysis to investigate our research questions.

#### 2.7.1. Descriptive Analysis

We used Microsoft Excel to generate simple descriptive statistics, such as the total number of recordings made in a month by each participant, etc., from the application usage logs.

#### 2.7.2. Statistical Analysis

We used STATA’s impute procedure to implement a multi-pattern regression-based algorithm to estimate PCS and MCS scores for the incomplete SF12v2 responses [57]. The imputation models were first generated as a function of the available SF12v2 items. Then, a distinct regression imputation model was fitted to predict each missing SF12v2 item, which ultimately, helped us calculate the MCS and PCS summary scores for all participants. Imputation was necessary because nine participants were missing up to five SF12v2 items, whereas the standard scoring algorithm required answers to all 12 individual items to calculate PCS and MCS summary scores.

The term “gain score” was used to refer to the difference between a participant’s post-study score and the pre-study score on each questionnaire (SF12v2 and OPQoL). To determine the statistical significance, we compared each participant’s pre- and post-study scores on each questionnaire using the paired Wilcoxon rank sign test [58], which is a non-parametric test that does not assume any underlying distribution of the data under evaluation. (For example, the *t*-test assumes normal distribution). The Wilcoxon rank sum test was used to compare between group differences because of the same reason. The non-normality of the data was confirmed using the Kolmogorov-Smirnov test. The level of statistical significance was set at *p* ≤ 0.05 for all measures in our analysis.

#### 2.7.3. Qualitative Analysis

The qualitative data consisted of end-of-study semi-structured interview transcripts. Three members of the research team independently performed a thematic analysis using an open coding approach, which involves first reading and re-reading the interview transcripts to familiarize with the discussed concepts and content. Open coding was then accomplished by segmenting participants’ comments into meaningful ideas and thoughts, and then describing them in a single word, or a short sequence of words (open codes). The results of open coding were a list of codes along with notes describing the content of codes and any striking observations and thoughts that are relevant around them. The researchers brought their code list and notes to a follow-up meeting, where these codes were compared and disagreements were resolved through discussions. The finalized codes were, then, collated under categories, which were then grouped under the research questions (considered as themes). The purpose of the qualitative analysis was to answer the following research questions.

- What benefits of the intervention were perceived by the participants?
- What barriers and challenges of the intervention were perceived by the participants?

## 3. Results

We present statistical analysis followed by the qualitative analysis results in the following subsections.

### 3.1. Application Usage

We used the participants’ usage of the goals module as a proxy to rate their overall usage of the eSeniorCare and gaming apps. (This was suitable because not all participants were using the medication management component of the application). A metric called Tracking activity (TrA), that is, the number of days in a month on which goals were tracked, was used to assign a frequency of use rating to each participant. Participants whose mean TrA was greater than nine and who had no missing month of tracking were considered frequent users. Participants with TrA less than five and who had missed more than two months of tracking were considered infrequent users. Thirteen participants were classified as frequent users and the remaining twelve participants were classified as infrequent users. The cut-off points for TrA and missing months were based on the analysis of the interaction logs, that is, application usage patterns.

### 3.2. SF12v2 Component Summary Scores

The SF12v2 MCS and PCS summary scores range from 0 to 100. Higher scores correspond to better mental and physical health performances [59]. Specifically, MCS scores below 42 are suggestive of ‘clinical depression’ [59], whereas PCS scores below 50 suggest sub-optimal physical health functioning.

#### 3.2.1. MCS Score

Pre-study MCS scores ranged from 30.31 to 68.03 (median = 57.99), with the MCS score of one participant at 30.31 (below 42). The post-study MCS scores ranged from 40.59 to 69.72 (median = 57.53). The Kolmogorov-Smirnov normality test confirmed that the gain scores were non-normally distributed (D=0.50,p≤0.05). The one-sided paired Wilcoxon rank sign test demonstrated that the median post-test MCS score ranks were not statistically significantly higher than the median pre-test MCS score ranks. Even though the changes were not statistically significant, 17 participants demonstrated improvements in their MCS scores, including the participant who had scored below 42 before the study. Two participants dropped their scores by more 10 points and one participants dropped by 5 points. The remaining demonstrated small decrements.

**Figure 4:**
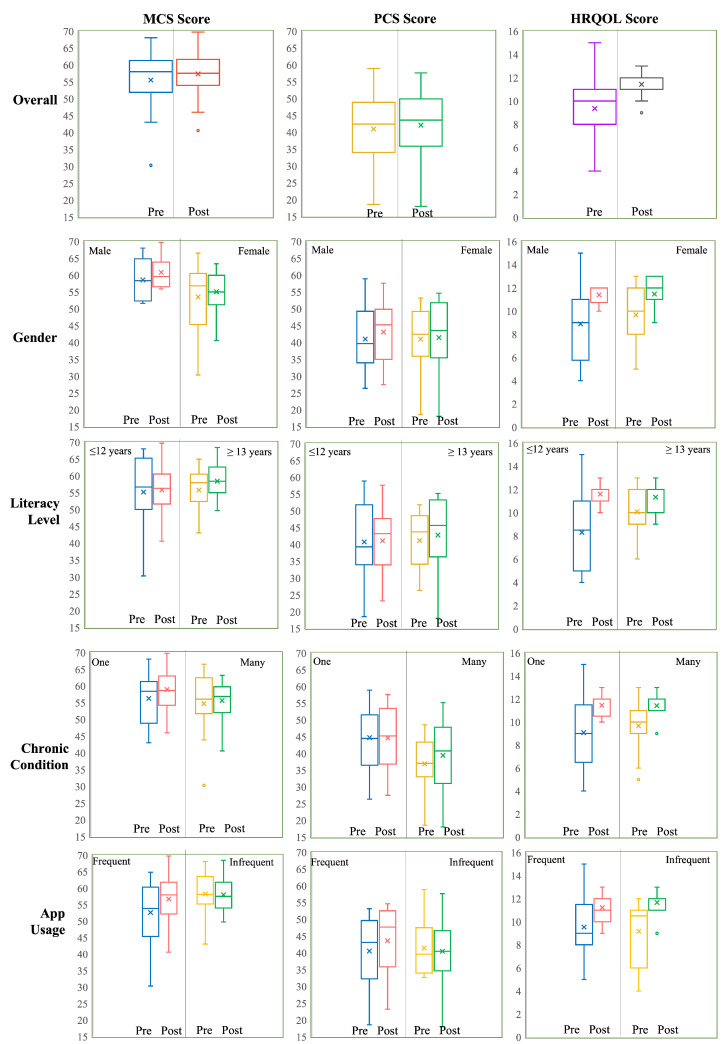
Pre and Post MCS, PCS and HRQoL score distribution according to various demographics. (x within the box is the mean and line is the median).

We also analyzed MCS scores based on gender (males and females), frequency of intervention use (infrequent and frequent), number of chronic conditions (one and many), and years of education (≤12 years and ≥13 years). Most groups experienced a small increase in their post-study median MCS scores, except for females and infrequent app users (Figure 4). The median score of females dropped by 1.83 points while that of infrequent users dropped by 0.52. To conduct paired comparisons, non-normality was first confirmed for each group. The Wilcoxon rank sign test was then conducted and it demonstrated that the post-test MCS score ranks (median = 58.19) of high literacy group were statistically significantly (indicated by *) different from their pre-study MCS score ranks (median = 57.53) (V=21, p=0.02877*). All other comparisons confirmed that the pre and post-study median MCS score ranks were not significantly different, statistically.

We also conducted unpaired comparisons of gain scores, that is, male versus female, infrequent versus frequent users, one versus multiple chronic conditions, and low-literacy versus high-literacy participants. Tests of non-normality were first done to ensure non-normality of the data sets. The Wilcoxon rank sum tests confirmed that the median MCS gain score ranks of the compared groups were not statistically significantly different from each other (Figure 4). In other words, no group had significant improvement in its mental health by the end of the study, when compared to each other.

#### 3.2.2. PCS Score

The overall physical health functioning of the majority of the participants was low, that is, ł 50. The pre-study PCS scores ranged from 18.54 to 58.89 (median =42.42). Five participant pre-study PCS scores were at and above 50. The post-study PCS scores ranged from 18.02 to 57.62 (median =43.58). The Kolmogorov-Smirnov normality test confirmed that the PCS gain scores were non-normally distributed (*D* =0.54, *p*≤0.05). The Wilcoxon rank sign test demonstrated that the post-study median PCS score ranks were not statistically significantly higher than the pre-study median PCS score ranks (paired one-sided Wilcoxon rank sign test: V=126, p=0.3388). Despite this, seventeen participants demonstrated improvements in their PCS scores by the end of the study. Out of these seventeen participants, six participants scored higher than 50. Three of these six participants had pre-study PCS scores below 50, suggesting a better physical health performance after the study. Two participants’ PCS scores fell below 50. Not all participants who improved their PCS scores also improved their MCS scores.

There was an increase in the median PCS scores across all groups by the end of the study (Figure 4). However, only the improvements in the post-study PCS score ranks (median=47.56) of frequent eSeniorCare users were statistically significantly higher than their pre-study PCS score ranks (median=44.01) (V=13, p=0.04546*). All other within-group changes in PCS score ranks were statistically insignificant.

Between-group (male versus female, frequent versus infrequent users, one versus multiple chronic conditions, and education ≤12 years versus ≥13 years) comparisons were also made using the Wilcoxon rank sum tests, after confirming non-normality with the Kolmogorov-Smirnov. None of the compared groups demonstrated statistically significantly differences in their median gain scores.

### 3.3. Health-Related Quality of Life

The HRQoL scores can range from 4 to 20, with higher scores indicating a higher health-related quality of life. At the onset of the study, the HRQoL scores ranged from 4 to 15 (median=10). Twenty participants scored below 12, indicating that the majority of the participants were experiencing below average health-related quality of life. The post-study HRQoL scores ranged from 9 to 13 (median=12). A total of seventeen participants increased their HRQoL scores and twelve scored above 12 by the end of the study, which shows that there was an overall improvement in participants’ perception of their HRQoL. Five experienced a decline in their HRQoL scores. The group of participants who had increased their post-study HRQoL scores was not the same as the one that had increased their PCS and MCS scores.

The Kolmogorov-Smirnov test confirmed non-normality of the HRQoL gain scores (*D* =0.524, *p* ≤0.05). The paired two-sided Wilcoxon rank sign test showed that the median post-study HRQoL rank scores were statistically significantly greater than the median pre-study HRQoL rank scores (V=41.5, p=0.005856*).

All groups showed improvements in their median HRQoL scores at the end of the study (Figure 4). The paired two-sided Wilcoxon rank sign test showed that the post-study HRQoL score ranks were statistically significantly higher than the pre-study HRQoL score ranks for males (V=6, p=0.03164*), low literacy group (V=7.5, p=0.0050307*), one chronic condition (V=10, p=0.04407*), and frequent eSeniorCare users (V=3.5, p=0.009743*). All other comparisons were statistically insignificant.

The between-groups comparisons, that is, male versus female, frequent versus infrequent users, one versus multiple chronic conditions and ≤12 years versus ≥13 years of education, were also made using the Wilcoxon rank sum tests, after confirming non-normality with the Kolmogorov-Smirnov test. All comparisons were found to be statistically insignificant.

### 3.4. Preferences

**Figure 5:**
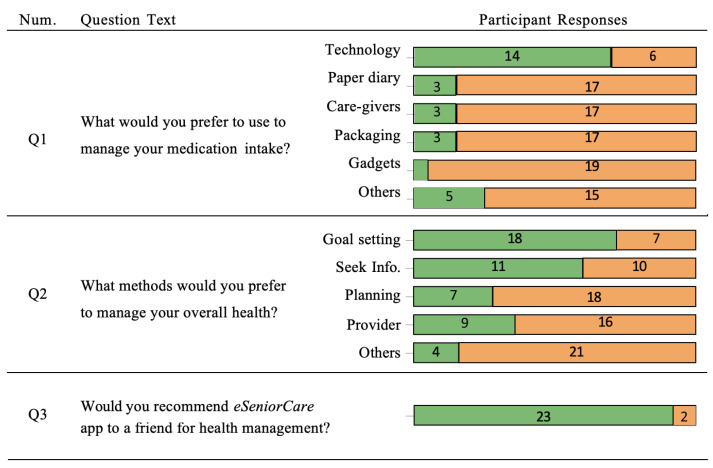
Questions and response trends for the Short Survey (Notes. Green color segment denotes ‘Yes’ while Orange denotes ‘No’ to the indicated option. The number values in the colored bar segments denote the number of participants who gave the corresponding response, while the length of the colored segment denotes the proportion of participants who agreed with the corresponding choice.)

The results of the short survey conducted during the semi-structured interview are summarized in Figure 5. Responses were collected as yes/no. Only participants who were on medication (n=20) answered the first question. The majority of participants who took medication (n=14 out of 20) or 70% indicated that they would prefer to use a technological solution to manage their medications and intake reminders. (n=18 out of 25) or 72% of participants stated that they would prefer to manage their overall health using a goal-setting tool. Other popular approaches included information seeking from various sources including providers. Overall, (n=23 out of 25) or 92% of participants agreed that they would recommend the eSeniorCare app to a friend because they had found the app beneficial for managing their health issues.

### 3.5. Perceived Benefits

Below, we describe the themes that were identified through our qualitative analysis. The descriptions include the actual comments made by different participants. Attributions are made with the help of participant’s ID, given in Table 2.

#### 3.5.1. Motivation to Care

Many participants thought that the eSeniorCare app had improved their sense of motivation and helped them redirect their focus back to their health. P131 explained that she had ignored her health for a long time but when she came across eSeniorCare, she realized that there were many things that she needed to work on. Therefore, she decided to try out the app by setting goals for herself and tracking them regularly. P98 indicated rewards from the app motivated her to continue her healthy habits. P93 further describes,

“It does give you a sense of motivation because once you use the app, you have the things that particular plan when you set the goal you want to accomplish and with the fast pace of life, it is so easy to get distracted from those things and having a tablet would allow you to get back on track. If you are missing out on one area or goals that you set, having the tablet would remind you to remember to set this goal, even though you might have gone outside, you can easily get back on.”

Participants found that the app increased their accountability towards their goals, by providing them with tools to accomplish those goals. P99 explained that she used the app to create goals and set reminders, which helped her become more organized and responsible. She knew that if she were not going to listen to the app, she would ignore her health. P101’s comment further explains this:

“It has been excellent as far as my medication is concerned. I had gone to a point where I was tired of taking medicine every day and was like not going to do this; so tired of taking this medicine every day. Sometimes, in my mind, I think it would be just okay if I do not take my medication today. Then, the alarms go off, and you are like ‘Okay; it is time for my medication so better to take it now.’”

This theme clearly demonstrates that the eSeniorCare application was able to change participants’ behaviors. The participants’ experiences with the application highlighted that they were able to incorporate it into their lives and use it to solve their problems.

#### 3.5.2. Awareness of Actions

The participants’ comments suggested that different features of the eSeniorCare app increased their awareness of their own health. For example, when P101 reflected on her medication intake patterns with the app, she realized how badly she ignored her health when she went into her depressive spell.

“It has been very instrumental in helping me see that my medication is very important and maintain a healthy lifestyle because I suffer from depression and I can be sitting in the house, depressed and might have that kind of spell for a week or so. When I am depressed, I want to do nothing. I will clean and only do the necessities. Sometimes, I would take my meds and sometimes I would not, but it [eSeniorCare app] helped me understand it.”

Another participant thought that the eSeniorCare app prevented her from mistakenly taking too many medications.

“It made me more aware of my medications. I would not have any problem getting involved with this app, as it is important to track my medications. So, I do not take the same thing more than times that I am supposed to because if I did that, then that could create other medical problems. Thus, it makes a person more conscious of how much medication they are taking. Are they following the medical diagnosis that the doctors have set for them?” (P101).

Furthermore, participants used the eSeniorCare app to confirm that they had already taken their medication.

“It helps me confirm I have taken my medications. The alarm goes off at 9 am and 9 pm. However, I have already taken it; it is right here on the tablet. The tablet is an additional confirmation” (P83).

With respect to goal setting, the participants used the app to reflect on their lives, and identify areas of their lives that needed attention. P95 stated,

“It was necessary. This helped me monitor how I spent my time. It told me where I was going. I can visualize my problems. Am I on or off course.”

P98 referred to the app as a *‘necessary exterior’*, which helps sum up this theme. Participants required a tool that could help them reflect on their lives as outsiders and identify areas in which they needed to direct their efforts and attention.

#### 3.5.3. Better Communication with Providers

The participants used the application as a communication tool in various health-related contexts. Participants reported that they appreciated having medication information on their tablets. They explained that the tablet was easy to carry and that it was always with them wherever they went. In particular, when they visited their providers for medical appointments or went for lab tests, bringing their tablets along was natural and intuitive, beecause all the information they needed for these interactions was already stored in their tablets.

“I love my medications on it. When I go to the hospital, they ask you what medications you are on? Here it is on eSeniorCare. Do I carry my prescriptions? No! I present pictures of my meds to the doctor. They appreciate this. They really like the app.” (P110).

Having a tablet as an information source and communication tool improved participants’ interactions with their providers. Participants explained that providers no longer had to expend extra time and effort to locate the prescription information. This meant that participants had more time to talk about their health problems and seek advice from the doctors. Moreover, they could explain their problems using their data on the app. For example, participants showed their medication intake records to their providers as well as their goals and tracking histories. This helped the providers better understand the participants’ problems and, hence, participants received better care at the end.

“The doctors like it when they can easily see the doses, what you are taking, and how you have taken. Doctors do not have a record and they can only go by what we have told them. It [eSeniorCare] has a record of what you did, are you taking your medicine on time and refilling regularly or not. This makes it easier for doctors because they do not have to look up and ask questions” (P96).

The comment below is an example of how a participant shared her goal data on the application with her physician and sought advice on further improving her health.

“The app is part of my life. I showed my doctor what I was doing. He was so impressed and said that you were so organized. He told me I could also set a goal to eat more fruits and vegetables to control my depression” (P98).

Participants used the eSeniorCare to share their data with their providers which helped them receive better care. Moreover, the application turned out to be a useful tool for providers.

#### 3.5.4. Self-Management Support

Participants who had previously used paper-based systems to track their medications and activities explained that the technology had eased many aspects of self-management by automating it. P95 used to track her medications in a diary, but when she started using eSeniorCare, she realized that the technology was more convenient and required less work. For similar reasons, the medication reminders generated by eSeniorCare were very appealing to P108.

“I used to track using paper. I can replace it with this tablet because when the reminder comes on, you know it is the time to take your medications. I prefer the tablet as it automatically provides reminders and tracks it.”

Some participants had never used paper-based solutions to solve their health management needs. This was because they found them too tiresome and time consuming. Moreover, some participants had some physical limitations, and they found that the technology is more convenient and easier to use than paper.

“When I was taking my insulin, it used to come with little diaries that you could use to write down. I can not write those down now as sometimes my nerves are so sore that writing becomes problematic. After this, I do not plan to go back to paper,” (P101).

This theme indicates how eSeniorCare was able to successfully support the self-management needs of study participants. It is important we continue to investigate how to reduce the burden of self-management with the help of mobile applications. The target population will have several physical limitations, and innovative designs are needed to circumvent these challenges.

#### 3.5.5. Improved Treatment Adherence

Participants found that the eSeniorCare app helped them become compliant with their prescriptions. In particular, several participants reported that ever since they started using the app, they had not missed their medications. The application kept them on track with their treatment goals even when they were busy and unaware of their slip ups.

“Sometimes I forget to take my medications, when I am at the hall, doing my worship. Then, alarm go off. I like the reminders, they are good,” (P95).

Another participant had started a medication regimen a few months before the start of the study. He was still not used to taking his medications on time when he first enrolled, but, after the study, he thought that the app had been very helpful in terms of changing his medication intake behavior.

“I like it because it reminds me of my medications. I have never taken medications in my life, so I was not in the practice of taking medications. The app reminds me,” (P98).

Some participants who had always meticulously followed their medication regimen felt that they sometimes needed reminders to take their medication. Many participants explained that they had started to forget more often without realizing it at first. They explained that they now depend on eSeniorCare to stay on track with their medication.

“First, I thought I do not need it. I take my medicine on time every day. But now I have started to forget. Sometimes, when I am doing something in the kitchen, I forget it. Then, the alarm goes off and you know it is time to take medications” (P115).

“Technology helps me keep up with my medication. My alarm goes off when it is time to take the medication. I am dependent on my alarms. If I do not hear the alarm, I do not take my medication” (P82).

Similarly, during the study, some participants set various goals for themselves, such as losing weight and quitting smoking. They explained that the app’s reminders about completing goals helped them achieve their goals. One participant explained this as follows:

“One of my goals was to lose some weight. If I do not get up and move, then I am not going to lose weight. I set a goal on the app to go to the gym and that [the eSeniorCare app] made me more accountable” (P110).

While the comments in this theme demonstrate how the eSeniorCare application helped participants pay close attention to their health, many participants also explained that they actually changed their behaviors because of the support they received from the application. For example, one participant quit smoking and another started going to the gym more frequently. Ultimately, treatment adherence is about changing behaviors. If people do not have the support to adhere to their goals, behavioral changes will be very difficult.

#### 3.5.6. Mental Stimulation

Participants reported that playing the games on their tablets provided mental stimulation. Many participants indicated that they were experiencing some mental health benefits. For example, some participants reported that they had experienced an improved ability to recall and memorize information. Others believed that playing on a tablet helped them think and solve problems more quickly.

“I like tablet games. They are making me think quicker” (P108).

Participants mentioned several other cognitive benefits of playing games. Some participants thought that playing games was helping them improve their hand and eye coordination. Some indicated it helped them become alert and attentive.

“It is good for the eyes too. It keeps you awake and it keeps you looking. It helps you enjoy. It is beneficial” (P100).

Clearly, there seem to be several short-term cognitive benefits to playing tablet games. Different benefits were associated with different tablet games. It is better to provide participants with a diverse set of games so that they can receive multiple cognitive effects.

### 3.6. Perceived Barriers

While there were several perceived benefits of using eSeniorCare, a few participants mentioned the following as barriers that could hinder app adoption:

#### 3.6.1. Misaligned Needs

Some parts of the application did not align with a few participants’ needs. For example, five participants did not have many medications, so they did not find the medication management feature to be beneficial.

“It did not help with the medications. I do not have enough medications to where it gets to like” (P102).

Other participants wanted to track certain health indicators such as blood pressure, pain levels, a functionality that was not explicitly built into the eSeniorCare app.

“If you could figure out how to put blood pressure on it, that would be key for people checking their blood pressure in the morning and at night. It will be more interesting because people want to know their lifeline because your blood pressure is your number one lifeline” (P133).

This theme demonstrates participants’ willingness to use the application for health management. Moreover, it demonstrates some of the challenges that participants faced when applying the application to solve their problems. For example, participants could have tracked their blood pressure by setting it as a goal in the app, but this was not immediately obvious to them. This theme enforces the need to design flexible and extensible user interfaces, which is one of our design goals for eSeniorCare.

#### 3.6.2. Lack of Trust

There was some hesitation in using the eSeniorCare app. Some participants did not feel comfortable tracking their health data only on the app, they also wanted to use paper. They indicated that the paper was more reliable, but the technology was more convenient. They feared that because of technological failures they would lose any information that they had stored in the app; they did not want to rely soley on the eSeniorCare app.

“I do both because tablet is good to have. I am still a paper-pencil girl. Together they are awesome. Right now, I do it through the tablet. However, there are times when I think I would track with a pencil and paper. I would track the same thing in both places, too many glitches of technology. I once saw a small glitch shut down airlines, so I am likely going to keep both” (P131).

Lack of technical familiarity and existing behaviors can hinder the adoption of technology in the target population. The participants were aware that we were backing up their data on the central server, but additional assurances were needed to be built into the app design. Moreover, even though, participants thought that they should track data in two different places, no one actually ended up tracking their data in two places. Less time and effort with tracking with mobile applications will help win over more users.

#### 3.6.3. ‘Not-for-Me’ Attitude

A few participants did not want to use paper or technology because they believed that they were already well aware of their activities. Other participants considered themselves self-sufficient and did not consider the need to have an external source to help them manage their lives.

“I do not need reminders. I am pretty smart. I wake up, I go to work, I go to the library, and cook supper. I am at the top of my medications. I am constantly aware of time. No alarms help. I know when it is time to take the med. I can take it without looking at time” (P91).

Participants who found eSeniorCare beneficial had similar sentiments before they used the application, but their views changed towards the end of the study. This theme mainly reflects the sentiments of those participants who might not have used the eSeniorCare app enough to change their views and of those who might perceive themselves to have stronger internal focus of control.

#### 3.6.4. Tracking Burden

A few participants were not interested in tracking their health data because they thought that it required effort, commitment, and time. They wanted to avoid doing the extra work. There was also a belief that tracking was not necessary to adopt new behaviors. Nonetheless, participants thought that the app was giving them the direction and momentum they needed to improve their health.

“Doing that [goals] and tracking them is hard for me because I am so used to being disciplined in doing things. I don’t think of tracking it. To me, it is normal. It’s like almost second nature” (P76).

“I changed a lot of things. I did not record them, but I looked at the visualizations [goal icons] and knew what I needed to do. So, it did help” (P133).

This theme shows that while participants valued some aspects of the app, they did not consider all of them to be useful. Moreover, they wanted to use the app to direct their attention towards a course leading to better health with minimal effort.

#### 3.6.5. Privacy Concerns

Concern for privacy was another issue that had some impact on participants’ decision to use the application. The main hesitation was unwillingness to share their health goals and private data. P85 told us that he did not want to place his information on the tablet and wanted to keep his life private. Mainly, participants were concerned that the AiP would find some of their goals, such as quitting drugs, objectionable and take measures to evict them from the housing facilities. This theme raises points to the importance of safeguarding patient’s privacy and incorporating designs to assuage invasion of privacy concerns.

## 4. Discussion

The results demonstrated that the use of the tablet application was correlated with improvements in self-perceptions of all three metrics of interest, that is, mental health, physical health, and health-related quality of life (Figure 4). However, only the post-study median HRQoL score ranks were statistically significantly higher than the pre-study median HRQoL score ranks across all participants. Moreover, within-group comparisons revealed that frequent eSeniorCare users experienced statistically significant improvements in PCS and HRQoL scores. Thematic analysis revealed that the use of the app motivated participants to care for themselves, increased their awareness about their health, improved their communication with providers, supported them in self-management of their health, improved adherence to treatment, and provided mental stimulation. They indicated that barriers that could influence their own or their peers’ adoption of the app in the future included lack of alignment with their needs, lack of trust in the app and ‘not-for-me’ attitude. The majority (*n* = 14 out of 20) or 70% of the participants reported that they would prefer to use technology for medication management, and (*n* = 18 out of 25) or 72% reported that they would use technology for health management. The majority (*n* = 23 out of 25) or 92% agreed that they would recommend the eSeniorCare app to their friends because of the benefits they experienced using it.

### Practical Implications

Existing studies [60,61] have demonstrated that mHealth applications do not significantly impact the physical health or physical activity levels of older adults. Moreover, existing studies indicate that the positive improvements produced by technology tend to attenuate over time. For example, in one study, tablet users who interacted with an embodied conversational agent were compared with non-tablet users [62]. Interviews were conducted at the end of two and twelve months of the study. Although the average number of steps recorded by the tablet group was greater than that recorded by the non-tablet group at both evaluation time points, there was a decrease in the average number of steps recorded from 2 to 12 months in both the groups. We did not conduct repeated evaluations; however, it is possible that the participants in our study experienced similar changes. Moreover, individuals tend to score lower on the PCS with age [63]. Based on these factors, it is not surprising that there was no significant change in PCS scores across all participants.

Research has demonstrated that older adults consider health an important indicator of their quality of life [56]. Therefore, we isolated participants’ HRQoL scores from their OPQoL scores. The participants’ HRQoL scores improved at the end of the study; moreover, improvements in HRQoL were statistically insignificant. Earlier studies did not support the claim that technology use directly enhances well-being among older adults [64,65]. Our study was different in that the earlier studies used computers and the Internet, whereas we used a touchscreen tablet (and specific applications). Research shows that older adults find touchscreen devices more convenient to use than laptops/desktops [66]. Therefore, it is possible that different technological media have varying impacts on older adults’ self-perceptions of well-being. Another distinction between our study and previous studies is that most studies did not measure quality of life outcomes over a long period (longitudinal) [67,68].

Studies have demonstrated that the effectiveness of technology-based interventions tends to be lower among older adults with low health literacy [62], which corresponds to low literacy skills [69]. We found that the low literacy group experienced statistically significant improvements in HRQoL scores. However, these results must be interpreted with caution because the number of participants in the two literacy groups (high and low) were unequal (10 and 15, respectively). There may also not be a significant difference in the literacy levels of participants with 12 and 13 years of education. Higher differences in literacy levels between the two groups may have provided more definitive results. Moreover, years of education is not always a true indicator of an individual’s literacy level.

It has been found that tracking medications encourages older adults to take their medications on time, which, in turn, helps them overcome medication non-adherence issues [70,71,72]. Moreover, research indicates that the ability to perform activities of daily living (IADL) can significantly improve older adults’ quality of life [73], and that the ability to perform IADL is directly related to one’s cognitive functioning [74,75]. Through our intervention, participants learned new tablet skills that are known to improve cognitive functions [74,75]. Moreover, they experienced social interactions with the AiP staff, who addressed the health and wellness needs of the participants throughout the study. In other words, our intervention consisted of all the components that have been found to have a significant impact on HRQoL; therefore, improvements in participants’ self-rating of their HRQoL have a strong basis.

Both frequent and infrequent eSeniorCare app users demonstrated improvements in PCS and HRQoL scores. However, only improvements in the frequent eSeniorCare users’ scores were statistically significant. The themes identified from qualitative analysis helps us understand some positive impacts of eSeniorCare on participants’ lives. Especially, participants mentioned that the app improved their ability to self-manage and stay compliant with their treatment. In addition, the app improved their awareness about their actions and overall mental health. Moreover, research has demonstrated that goal-setting can promote adherence to physical health [42]. However, merely analyzing the usage frequency of the goal-setting feature is not sufficient to understand the relationship between the usage of the eSeniorCare app and improvement in health functioning scores. A detailed analysis of the nature of tracked goals would provide a more accurate snapshot of physical health, and clarify the relationship between physical health and eSeniorCare usage [42,43]. For example, activities such as exercise, walking, quitting alcohol or cigarettes, and weight loss affect physical health. Further analysis is required to draw conclusions regarding this finding. In any case, according to Kolotkin et al. [76], HRQoL improvements are more meaningful when individuals start with low HRQoL scores at the baseline. Therefore, increases in the HRQoL scores point to a possibly positive impact of mHealth interventions in the study participants.

With a few exceptions, prior studies have not used specific behavior change strategies, nor have they provided specific details of the behavior change techniques used [23]. We used a goal-setting strategy to influence the participants’ physical health, and our findings suggest that this strategy is acceptable for the target population. Moreover, as in other published studies, participants in our study indicated that they would only use and accept technology, if they perceived it to be useful to their lives [26,77]. Moreover, participants explained that they would not use an app; if it did not meet their needs, they did not trust it, or it did not align with their values. In the future, these challenges can be addressed by improving the app design or increasing participants’ comfort with and knowledge of the technology.

Published studies have reported that learning technology skills has a positive impact on the mental health of older adults [74,75]. Although there was a negligible change in median MCS scores over the course of the study, one participant with the lowest MCS score increased it by 10.28 points, suggesting that technology use might be more beneficial for people with poor mental health functioning. Moreover, frequent eSeniorCare users also experienced an improvement in their median MCS scores, and this group had lower median score compared to the infrequent eSeniorCare users. It is possible that participants with above-normal mental functioning may not perceive drastic improvements in their mental health through technology use over a short period. Studies show that, with age, people tend to score lower on the physical health scale (PCS) but higher on the mental health scale (MCS). As there are systematic age differences, it is important to compare these scores with those reported in the literature. For example, Linde et al. [78] report PCS and MCS scores within 56–57 for adults above 50. The mean MCS score of participants in our study is close to this range, but their PCS score is much lower (pre: 42.42; post: 43.58), which makes it difficult to make comparison. Nonetheless, there was an improvement in participants’ mean PCS score at the end of the study, suggesting a possible positive impact of mHealth interventions on physical health. Shah and Brown [79] report mean PCS 41.90 and mean MCS 53.10 for mean age 74.10 years (SD=6.19). The study participants (n=8) with mean age 74.0 years (SD=5.5) scored higher on their PCS (pre:45.92; post:47.89), but similarly on their MCS (pre:53.05). However, the post-study MCS (post:56.43) score was higher [79,80]. In certain patient populations with mean age 70 years, a change of 3.9 and 2.8 in PCS and MCS scores, respectively, are considered to be clinically meaningful [81]. Based on this evidence, we conclude a possibly positive impact of mHealth interventions on the mental health functioning of people within this age range.

Current evidence regarding the use and acceptance of health management technologies among older adults is mixed. D’Haeseleer et al. reported that older adults in their study demonstrated a lack of interest in adopting self-management technologies due to accessibility barriers and the need for relatives to provide support [82]. Other researchers suggest that older people would accept a technology as long as it is designed around their needs [83]. The findings of our study indicated that participants found the application to be beneficial, and indicated that they preferred to use technology-based solutions as opposed to paper-based ones for self-managing their medications and activities. Our study adds to the body of literature that supports the position that older adults accept digital technologies for self-management of their health.

Researchers are exploring the possibility of using big data management mechanisms to provide care services to older adults and help them achieve Successful Aging [26,84,85]. Such approaches involve digital equipment and technological solutions at an older person’s home to acquire real-time data for predictive and optimized health service delivery. Given that the present study has helped us establish the usability and feasibility of mHealth apps in the target population, we are planning to incorporate big data approaches within the eSeniorCare platform to curate services to support the medication intake, goal management and mental health needs of older adults. A critical aspect of this endeavor is the investigation of ethical issues to ensure that the system aligns with the needs and values of the target population. The participants in our study demonstrated the need for privacy, reliability, minimized tracking, and personalized features. These needs should be further investigated to ensure the success of these systems.

## 5. Limitations

This study has several limitations that can have an impact on the generalizability of the findings. This study was based on only 25 participants from two low-income independent living facilities in a rural area of the United States. Although this number helps us draw statistically significant conclusions from the tests (Wilcoxon rank sign and Wilcoxon rank sum) used in this study, the results are still under-powered. Moreover, the samples for the between-groups tests were even smaller (10–14) and unequal in sizes. These conditions reduced the statistical power of the comparisons. The specific conditions of the study sites, such as neighborhood, availability of the Internet, provision of other services, attitudes of independent living staff, and coordination between the residents, are some other factors that might have influenced the study findings. Moreover, only 6.25% of the residents of both facilities participated in this study. These older adults also participated more broadly in other activities organized at the study sites. Therefore, the results reported here cannot be generalized to other locations, countries, and individuals; the results should be viewed as preliminary and indicative only.

African Americans as a race were over-represented in this study. Although slowly changing, cultural and lifestyles nuances still exist in this group that need to be considered [86]. Moreover, research suggests that African Americans, especially, those with low SES are at a higher risk of certain chronic conditions, such as diabetes [87]. Therefore, the findings of this study may be more representative of certain racial groups. Therefore, future studies should, therefore, aim for a more balanced racial composition.

Another possible limitation of the study is that the tablet was provided as a remuneration for participating in the study. It is possible that the participants’ comments were influenced by this compensation. However, this possibility seems very slim, since a new model of the tablet was already available in the market sometime during the study. Moreover, the tablet used in the study was inexpensive and several participants had told us that they would have preferred to have a tablet with a bigger screen.

The findings of this study were mostly based on self-reported quantitative and qualitative data. We also did not use any objective measures to determine the feasibility of eSeniorCare on health outcomes. For example, we did not measure changes in step counts (for increases in physical activity), sleep time (for improved sleep), or decreases in medical costs (for improved physical health), etc. Further rigorous impact research, including clinical efficacy, cost-effectiveness, sustainability, etc. is still needed in addition to the reported findings. Future studies should use both subjective and objective measures to study the long-term impact.

## 6. Conclusions

The objective of this research was to test the feasibility of the eSeniorCare app to improve self-perceptions of Successful Aging in community-dwelling older adults with low-SES. The study findings indicate that the tablet use is correlated with improvements in the participants’ self-perceptions of physical health and health-related quality of life. In addition, the majority of participants reported several perceived benefits of using eSeniorCare (tablet-based app), such as motivation to care for themselves, increased awareness of their actions, and improved treatment adherence. These perceptions suggest that participants were satisfied with the eSeniorCare application. Some barriers (e.g., lack of trust, privacy threats, and lack of features of interest) may negatively impact app usage. Nonetheless, the participants indicated that they would be willing to use the app if future benefits surpassed the existing limitations. Overall, we conclude that mHealth apps are feasible for long-term use and perceived to be beneficial by older adults with low SES. They may also simultaneously enhance multiple domains of Successful Aging in community-dwelling older adults. We are planning another study to determine the long-term impact of eSeniorCare app use in older adults by collecting objective measures of Successful Aging.

## Figures and Tables

**Table 1:** Tablet Training Workshops-Topics and frequency during first three months.

Workshop No.	Workshop Topic	Total Times
1	Tablet basics (hardware buttons, tablet settings, etc.)	2
2	Tablet apps (calendar, reminders, weather, etc.)	2
3	eSeniorCare App	2
4	Communication apps (Gmail, Skype, Facebook)	2
5	Gaming apps (shape, image and word puzzles)	4

**Table 2:** Participants’ demographics (PID = Participant Identifier, ${}^{‡}$ = Prescribed Medications, AA = African American, WC = White Caucasian, ME = Multi-Ethnic, and Alone = Living Alone).

PID	Age (Years)	Gender	Education (Years)	Race	Income	Alone?
P74	65	Female	12	AA	0–$10 K	Yes
P76 ${}^{‡}$	62	Female	14	AA	10–$20 K	Yes
P80 ${}^{‡}$	72	Female	12	AA	10–$20 K	No
P82 ${}^{‡}$	58	Female	14	AA	0–$10 K	Yes
P83 ${}^{‡}$	69	Male	8	AA	0–$10 K	Yes
P85 ${}^{‡}$	64	Female	14	AA	10–$20 K	Yes
P88 ${}^{‡}$	72	Male	12	AA	0–$10 K	No
P89	61	Male	14	AA	10–$20 K	Yes
P91 ${}^{‡}$	66	Male	8	WC	20–$30 K	Yes
P93	60	Male	14	AA	0–$10 K	Yes
P94	59	Female	16	AA	0–$10 K	Yes
P95 ${}^{‡}$	56	Female	14	AA	0–$10 K	Yes
P96 ${}^{‡}$	64	Male	14	AA	10–$20 K	Yes
P98 ${}^{‡}$	66	Female	14	AA	0–$10 K	Yes
P99	72	Female	14	AA	0–$10 K	No
P100 ${}^{‡}$	64	Female	14.5	AA	0–$10 K	No
P101 ${}^{‡}$	57	Female	13	AA	0–$10 K	Yes
P102 ${}^{‡}$	79	Male	12	AA	0–$10 K	Yes
P107 ${}^{‡}$	75	Female	8	AA	0–$10 K	Yes
P108 ${}^{‡}$	83	Female	10	AA	0–$10 K	Yes
P110 ${}^{‡}$	65	Male	13.5	ME	0–$10 K	No
P115 ${}^{‡}$	73	Male	8	AA	0–$10 K	Yes
P131 ${}^{‡}$	61	Male	14	AA	0–$10 K	Yes
P133 ${}^{‡}$	59	Female	13	WC	0–$10 K	Yes
P189 ${}^{‡}$	61	Male	11	AA	0–$10 K	Yes

## Data Availability

The data set can be obtained by emailing the corresponding author.
